# The Role of a Selective P2Y_6_ Receptor Antagonist, MRS2578, on the Formation of Angiotensin II-Induced Abdominal Aortic Aneurysms

**DOI:** 10.1155/2020/1983940

**Published:** 2020-04-18

**Authors:** Xiao Du, Shilan Zhang, Qunyan Xiang, Jingyuan Chen, Feng Tian, Jin Xu, Xin Li, Yuansheng Tan, Ling Liu

**Affiliations:** ^1^Department of Cardiovascular Medicine, The Second Xiangya Hospital, Central South University, Changsha, Hunan 410011, China; ^2^Department of Gastroenterology, The Second Xiangya Hospital, Central South University, Changsha, Hunan 410011, China; ^3^Ministry of Education, Hospital of Hunan University of Traditional Chinese Medicine, Changsha, Hunan 410011, China

## Abstract

**Objective:**

The P2Y_6_ receptor has been shown to be involved in many cardiovascular diseases, including hypertension and atherosclerosis. The study is aimed at exploring the role of the P2Y_6_ receptor in Ang II-induced abdominal aortic aneurysm (AAA) formation in apolipoprotein E-deficient (apoE^−/−^) mice by using its selective antagonist.

**Methods:**

Male apoE^−/−^ mice were fed with high-fat diet and infused with angiotensin (Ang) II (1000 ng/kg/min) for 4 weeks to induce AAA or saline as controls. Mice were divided into four groups: normal saline (NS, placebo control) group (*n* = 8), Ang II+vehicle (Ang II) group (*n* = 14), Ang II-low dose MRS2578 (Ang II+MRS-16 mg) group (*n* = 14), and Ang II-high dose MRS2578 (Ang II+MRS-32 mg) group (*n* = 14). Daily intraperitoneal injection with vehicle or MRS2578 was pretreated one week before Ang II infusion. On postoperative day 10, aorta imaging of each group was taken by ultrasonography. After 4 weeks of Ang II infusion, the excised aortas were processed for diameter measurement and quantification of aneurysm severity and tissue characteristics; the blood samples were collected for measurement of the lipid profile and levels of cytokines. Verhoeff's Van Gieson (EVG) staining and immunochemistry staining were performed to evaluate disruption of the extracellular matrix (ECM) and infiltration of macrophages. Expression and activity of matrix metalloproteinases (MMPs) was measured by gelatin zymography.

**Results:**

Treatment with MRS2578 made no significant difference in AAA formation, and maximal aortic diameter yet caused higher AAA rupture-induced mortality from 7% (Ang II) to 21.4% (Ang II+MRS-16 mg) or 42.9% (Ang II+MRS-32 mg), respectively (*p* < 0.05). Consistently, the severity of aneurysm tended to be more deteriorated in MRS2578-treated groups, especially the high-dosage group. The ratios of type III and IV aneurysm were much higher in the MRS2578-coadministered groups (*p* < 0.05). Furthermore, histological analyses showed that administration of MRS2578 significantly increased infiltration of macrophages, expression of monocyte chemotactic protein 1 (MCP-1) and vascular cell adhesion molecule-1 (VCAM-1), and activities of MMP-2 and MMP-9 followed by aggravating degradation elastin in vivo (*p* < 0.05). However, the multiple effects of MRS2578 on the development of AAA are independent of changes in systolic blood pressure and lipid profiles.

**Conclusions:**

The present study demonstrated that administration of MRS2578 exacerbated the progression and rupture of experimental AAA through promoting proinflammatory response and MMP expression and activity, which indicated a crucial role of the P2Y_6_ receptor in AAA development. *Clinical Relevance*. Purinergic P2Y receptors have attracted much attention since the P2Y_12_ receptor antagonist had been successfully applied in clinical practice. Elucidating the underlying mechanisms of AAA and exploring potential therapeutic strategies are essential to prevent its progression and reduce the mortality rate.

## 1. Introduction

Abdominal aortic aneurysm (AAA) is a permanent, degenerative vascular disease, characterized by localized enlargement of the abdominal aorta exceeding the normal vascular diameter by more than 50% [1]. In spite of high mortality and morbidity, AAA is often asymptomatic in its earliest stage. Once the aneurysm is larger than 5.5 cm in size, surgical repair is the only applicable treatment to reduce the mortality of AAA even with a considerable perioperative risk [2]. However, most patients with AAA have relatively small aneurysms that are incompatible with surgical indications, and thus, effective drugs are needed to delay progression and prevent rupture of AAA [3, 4]. It has been established that the typically pathologic feature of AAA in both animal models and patients is chronic vascular inflammation [5, 6]. Available evidence suggests that it is associated with increased infiltration of macrophages in arterial walls driven by inflammatory factors, such as vascular cell adhesion molecule-1 (VCAM-1) and monocyte chemotactic protein 1 (MCP-1), and obvious degradation of the extracellular matrix (ECM) caused by activated matrix metalloproteinases (MMPs) and apoptosis of medial vascular smooth muscle cells (VSMCs) [1, 7–9].

Purinoceptors, playing a pivotal role in cardiovascular diseases, were firstly proposed in 1972 and divided into P1 receptor family and P2 receptor family [10, 11]. Purinergic P2Y receptors have been classified into eight diverse subtypes, including P2Y_6_ and P2Y_12_ [11]. The selective P2Y_12_ antagonist, AZD6140 or clopidogrel, can limit the formation and progression of experimental AAA via inhibiting the vascular inflammation or platelet activation [12, 13]. The P2Y_6_ receptor is widely expressed on macrophages, VSMCs, and endothelial cells [14, 15], all of which participate in the processes of both atherosclerosis and AAA. Growing evidence has confirmed that the P2Y_6_ receptor participates in atherosclerosis and hypertension by augmenting proinflammatory responses in macrophages or vascular contraction, respectively [16]. It is well established that the P2Y_6_ receptor is abundantly expressed in atherosclerotic lesions of low-density lipoprotein receptor (LDLR) knockout mice, and the knockout of the P2Y_6_ receptor will alleviate atherosclerosis and plaque inflammation [16, 17]. However, the potential role of inhibiting the P2Y_6_ receptor during the development of AAA has not yet been elucidated.

Thus, this study is aimed at evaluating the effect of MRS2578, as a selective P2Y_6_ receptor antagonist, in Angiotensin (Ang) II-induced AAA formation in apolipoprotein E-deficient (apoE^−/−^) mice. We found that MRS2578 treatment significantly promotes the progression and rupture of AAA. Inhibition of the P2Y_6_ receptor substantially increased the macrophage recruitment, activation of MMPs, and degradation of ECM.

## 2. Materials and Methods

### 2.1. Animal Model and Experimental Design Protocol

All animal experiments were approved by the ethics committee of the Central South University or the Institutional Animal Care and Use Committee at the Second Xiangya Hospital of Central South University.

In this study, we used Ang II-induced apoE^−/−^ mice (8 to 10 weeks old, male) to establish an AAA model as described previously [18]. ApoE^−/−^ mice in C57BL/6 background were obtained from the Jackson Laboratory (Sacramento, California) and maintained with a high-fat diet (C12108; Research Diets, NJ, USA) during the Ang II (1,000 ng/kg/min, Sigma, St Louis, MO, USA) perfusion. Mice were randomly assigned to four groups: normal saline (NS, placebo control) group, Ang II+vehicle (Ang II) group, Ang II-low dose MRS2578 (Ang II+MRS-16 mg) group, and Ang II-high dose MRS2578 (Ang II+MRS-32 mg) group. After being anesthetized with sodium pentobarbital (100 mg/kg), animals were subcutaneously implanted with ALZET miniosmotic pumps (Model 2004, DURECT Corp., CA, USA) containing Ang II or NS [19].

Injection of MRS2578, a selective P2Y_6_ receptor antagonist, was initiated 1 week before the implantation and continued daily during the Ang II perfusion. At the end of the study, all mice were sacrificed and the aortas were cleaned of blood by perfusing heparinized saline through the left ventricle.

After carefully removing the adipose tissue around the periaortic adventitia, aortas were subsequently perfused with 4% paraformaldehyde overnight and photographed for diameter measurements using Image-Pro software (Image-Pro Plus, EPIX Vision). By measuring the maximal outer diameter of suprarenal aortas, AAA was defined as ≥50% dilation of the suprarenal region compared with its normal region of aortas.

According to the severity of aneurysm, we classified AAA to four types: type I, dilated lumen without thrombus; type II, remodeled aneurysmal tissue with little thrombus; type III, a pronounced bulbous form of type II with thrombus; and type IV, multiple often overlapping aneurysms containing thrombi, as previously reported [19, 20].

### 2.2. Measurement of Haemodynamics and Ultrasonography

Systolic blood pressure (SBP) was measured by a noninvasive tail-cuff system (BP-2010A, Softron Biotechnology) at baseline and 7 and 28 days after Ang II infusion, as previously described [20, 21] . SBP data were recorded between 8 am and 10 am each time. The investigator was blinded to experimental groups to perform the measurements, while mice were randomly tested for 5 consecutive times.

After 10 days of Ang II infusion, aorta imaging of each group was performed in a blinded manner. The aortic size was measured by use of the ultrasound system (ACUSON S3000, Siemens, Germany) equipped with an 18 MHz transducer. Color Doppler examination was performed to detect arterial flow, as previously described [12].

### 2.3. Serum Analyses and Lipid Profile

Blood sample were collected from the angular vein with EDTA for lipid analysis. Serum TC, TG, HDL-C, and LDL-C were quantified on a Hitachi 7600-020 analyzer by a specialist who was blinded to this study, as previously described [22]. Plasma concentration of MCP-1 (ab100721, Abcam) and VCAM-1 (ab201218, Abcam) was determined using an ELISA kit, following the manufacturer's instructions.

### 2.4. Histological Analyses and Immunohistochemistry

Formaldehyde-fixed paraffin sections were cut in cross section (5 *μ*m) and then stained with hematoxylin and eosin (HE) for analysis of structural integrity, Verhoeff's Van Gieson (EVG) staining for corruption of elastin, and Mac-3 for infiltration of macrophages.

The severity of elastin degradation is semiquantified, as previously described (grade 1, no degradation; grade 2, mild degradation; grade 3, severe degradation; and grade 4, presence of aortic rupture) [12].

Briefly, the aortic sections were deparaffinized and hydrated in H_2_O and then incubated with primary antibodies against Mac-3 (1 : 100 dilution, BD, 550292), MCP-1 (1 : 500 dilution, Servicebio, G11199), VCAM-1 (1 : 200 dilution, Servicebio, G13336), MMP-2 (1 : 6000 dilution, Servicebio, GB11130), and MMP-9 (1 : 1000 dilution, Servicebio, GB11132) at 4°C overnight, followed by incubation with the corresponding secondary antibody for 1 h at 37°C. Then, the sections were dehydrated and images were obtained by a Nikon microscope equipped with a CCD camera. Immunostaining data of each section was randomly selected and measured by 2 different investigators in a blinded manner using the image analysis software (Image-Pro Plus, EPIX Vision).

### 2.5. Gelatin Zymography

Gelatin zymography was performed to evaluate MMP-2 and MMP-9 enzymatic activities in aortic tissues as previously described [23]. Briefly, an equal concentration of extracted protein from mouse aortas was separated on 8% acrylamide-SDS gel containing 1 mg/ml of gelatin at room temperature. Proteins were renatured in 2.5% (vol/vol) Triton X-100 for 3 hours and subsequently incubated at 37°C for 48 hours in developing buffer (50 mM Tris-HCl, 5 mM CaCl_2_, and 0.02% Brij-35). Gelatinase activity was showed as clear bands against a blue background after staining with 0.25% Coomassie Brilliant Blue. The intensity of bands for MMPs was quantified by Image-Pro Plus software.

### 2.6. Statistical Analysis

Data are expressed as mean ± standard error of measurement (SEM) for continuous variables and frequencies (percentages) for categorical ones. ANOVA and the Student-Newman-Keuls post hoc test were used to determine differences between the treated animals and the control and statistical significance. In vivo data were quantified using Image-Pro Plus software and presented as mean ± SEM. The sample size is documented in the respective figure legend. All analyses were assessed using the Statistical Package for the Social Sciences, version 19.0 (SPSS Inc., Chicago, IL, USA). Differences were considered significant at a *p* value of <0.05.

## 3. Results

### 3.1. MRS2578 Augmented Severity of AAA but Not the Incidence

**Figure 1:**
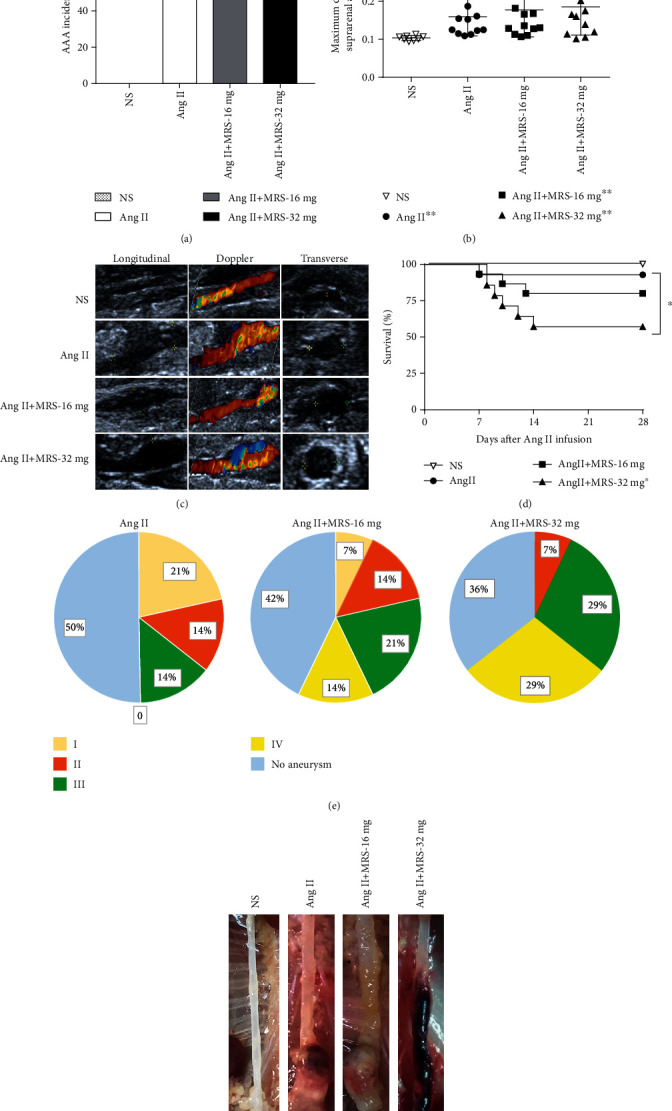
MRS2578 treatment exacerbates Ang II-induced AAA formation in apoE^−/−^ mice. (a) The incidence rates of Ang II-induced AAA in the NS, Ang II, Ang II+MRS-16 mg, and Ang II+MRS-32 mg groups (*n* = 14; *p* = 0.746). (b) Maximal abdominal aortic diameter in the NS, Ang II, Ang II+MRS-16 mg, and Ang II+MRS-32 mg groups (*n* = 8-14; ^∗∗^*p* < 0.01 vs. NS group). (c) Representative images of abdominal aortic lumen diameters quantified by ultrasound over 10 days of Ang II infusions in mice from each group. Longitudinal (left), Doppler (center), transverse (right). (d) The survival curve shows the survival rate in mice receiving Ang II (or NS) infusion only and Ang II cotreated with MRS2578 (*n* = 14; ^∗^*p* < 0.05 vs. the Ang II group). The log-rank (Mantel-Cox) test. (e) Severity of aneurysm in each group was categorized according to the classification scheme described previously (*n* = 14; ^∗∗^*p* < 0.01 vs. the Ang II group). (f) Representative images of aortas from mice treated with NS (left), Ang II (second from the left), Ang II+MRS-16 mg (center), and Ang II+MRS-32 mg (right). All values are shown as mean ± SEM.

To investigate the role of the P2Y_6_ receptor in AAA formation, 16~32 mg MRS2578, the specific P2Y_6_ receptor antagonist, or vehicle was administrated via intraperitoneal injection 1 week before Ang II infusion. Although there was an increase in the incidence of AAA with the elevated dosage of MRS2578 over 4 weeks of treatment of Ang II, the difference in the AAA incidence among the three Ang II perfusion groups did not reach statistical significance (*p* = 0.746; Figure 1(a)). Similarly, we also observed a significantly increased maximal diameter of the aortas in mice perfused with Ang II compared with the control group (*p* < 0.001; Figure 1(b)). Administration of MRS2578 led to an increase in the maximal diameter of the abdominal aorta, but there was no significant difference in the maximal diameter among the three Ang II perfusion groups (*p* > 0.05; Figure 1(b)). As shown in Figure 1(c), ultrasonography was used to measure the diameter of the suprarenal aorta at the early stage of AAA formation. We observed that the coadministration of the MRS2578 group led to further enlargement of the suprarenal aorta than that of the Ang II group at the end of 10 days of Ang II infusion, especially in the Ang II+MRS-32 mg group. Furthermore, only 7% (1/14) of mice of the Ang II group died as a result of AAA rupture, whereas 21.4% (3/14) of mice in the Ang II+MRS-16 mg group and 42.9% (6/14) of mice in the Ang II+MRS-32 mg group died within the initial 14 days. There was a significant difference in mortality among the three groups (*p*<0.05; Figure 1(d)). The aneurysm severity was also quantified according to the AAA classification system. With the increase in MRS2578 dosage, the percentage of type I or II aneurysm gradually decreased while that of type III or IV aneurysm gradually increased, and the difference in the proportion of each type of aneurysm among the three groups reached statistical significance (*p* < 0.05; Figure 1(e)). Representative photographs of the suprarenal aortas in each group were shown in Figure 1(f). Collectively, the above data suggested that MRS2578 may serve as an aggravating factor rather than a protective factor in AAA formation and rupture.

### 3.2. MRS2578 Administration Potentiated the Activities of MMPs and Degradation of ECM

**Figure 2:**
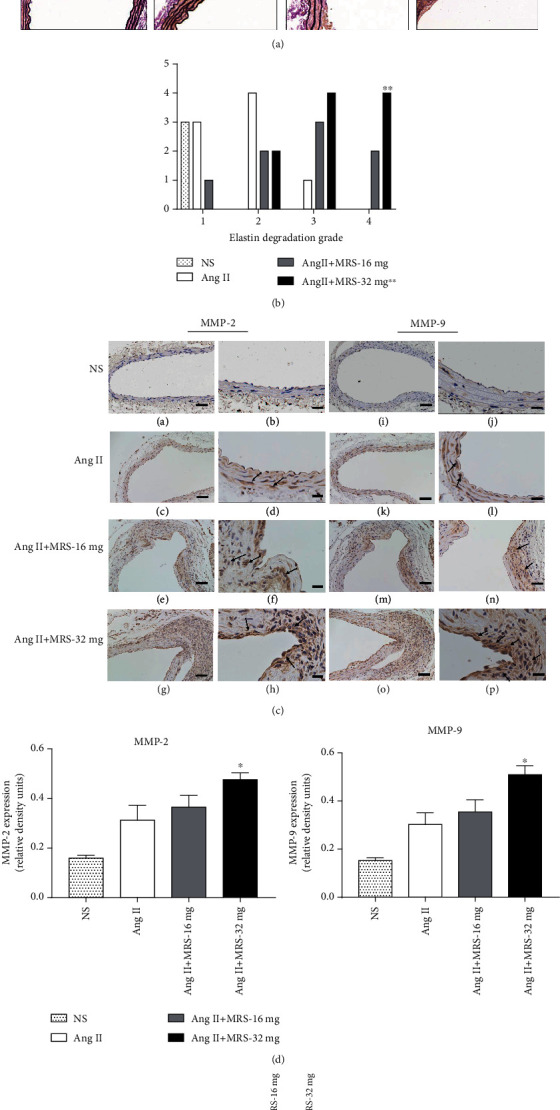
MRS2578 administration promotes elastin degradation and MMP-2 and MMP-9 expressions and activities in the abdominal aorta from Ang II-infused apoE^−/−^ mice. (a) Representative photomicrographs of EVG staining of aortic sections after a 28-day infusion of Ang II (or NS) with or without MRS2578. The upper line shown as 40x. Boxed areas are expanded to show representative high-power fields in serial sections (100x). (b) Semiquantitation of elastin degradation scores (^∗∗^*p* < 0.01 vs. the Ang II group). (c) Immunohistochemical staining showing expression of MMP-2 (A, C, E, and G, scale bar: 50 *μ*m) with its high-magnification images (B, D, F, and H, scale bar: 20 *μ*m) and MMP-9 (I, K, M, and O, scale bar: 50 *μ*m) with its high-magnification images (J, L, N, and P, scale bar: 20 *μ*m). (d) Densitometric quantification of expression of MMP-2 and MMP-9 (*n* = 3; ^∗^*p* < 0.05 vs. the Ang II group). (e) Gelatin gel zymography was used to detect MMP activity in aortic tissue extracts from different groups of mice.

As previous studies reported, vascular macrophage infiltration, overexpression of MMPs, especially MMP-2 and MMP-9, and subsequently destruction of ECM are critical events for AAA development and aortic rupture. To assess the effect of MRS2578 on the degeneration and destruction of the medial elastic layers, EVG staining and immunohistochemistry were performed. On the basis of quantitative analysis of elastin degradation described previously [12], fragmentation of ECM in the Ang II+MRS-32 mg group was severe than that in the Ang II group (*p* < 0.01, Figures 2(a) and 2(b)). Importantly, the expressions of MMP-2 and MMP-9 within the abdominal aortic wall of the two MRS2578 groups seemed greater than those of the Ang II group; however, only the difference between the Ang II+MRS-32 mg and Ang II groups reached statistical significance (*p* = 0.0193 and *p* = 0.0190, respectively; Figures 2(c) and 2(d)). Consistently, higher activities of MMP-2 and MMP-9 were detected in mice treated with both Ang II and MRS2578 through gelatin zymography, especially in the Ang II+MRS-32 mg group (Figure 2(e)), demonstrating that MRS2578 could contribute to rupture of AAA by accelerating disruption of the medial layer via increasing the expressions and activities of MMP-2 and MMP-9.

### 3.3. MRS2578 Accelerated the Infiltration of Macrophages and Vascular Inflammation in Ang II-Induced AAA

**Figure 3:**
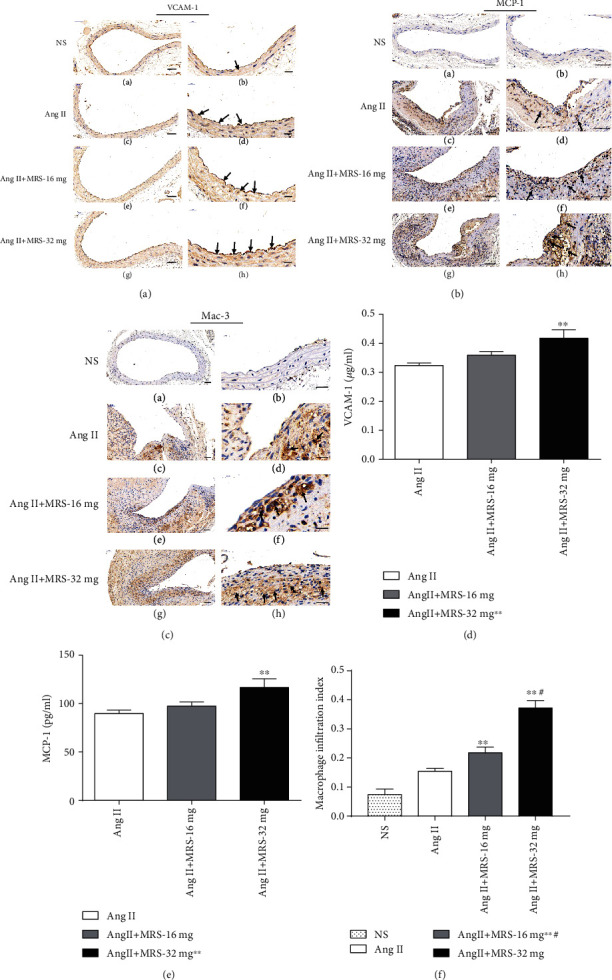
MRS2578 contributes to secretion of adhesion molecules and chemokines and macrophage infiltration into the aorta after Ang II infusion. (a) Representative images of immunohistochemical stains for VACM-1 in original magnification (left, 200x, scale bar: 50 *μ*m) and amplified images (right, 800x, scale bar: 20 *μ*m). (b) MCP-1 levels in abdominal aortic segments (left, 200x; right, 400x, scale bar: 50 *μ*m). (c) Representative photomicrographs and Mac-3 immunostaining in abdominal aortic segments from each group (left, 200x, scale bar: 50 *μ*m; right, 800x, scale bar: 20 *μ*m). Arrowheads indicate the positive cells. Expression of (d) VCAM-1 and (e) MCP-1 in plasma measured by ELISA (*n* = 8~10; ^∗^*p* < 0.05 and ^∗∗^*p* < 0.01 vs. the Ang II group). (f) Quantification of Mac-3-immunopositive cells (^#^*p* < 0.05 and ^∗∗^*p* < 0.01 vs. the Ang II group; ^#^*p* < 0.05 vs. the Ang II+MRS-16 mg). Data represent mean ± SEM; *n* = 5 per group.

Chronic inflammation of vasculature is also responsible for AAA formation. Therefore, the expressions of VCAM-1 and MCP-1, both of which contribute to leukocyte infiltration, were measured by immunohistochemical methods. Notably, the expression of VCAM-1 on the surface of the endothelium as well as that of MCP-1 in the media and adventitia was dramatically enhanced in mice treated with MRS2578, especially in the Ang II+MRS-32 mg group (Figures 3(a) and 3(b)). Likewise, plasma levels of VCAM-1 and MCP-1 in the Ang II+MRS-32 mg were also significantly higher than those in the Ang II group (*p* = 0.0142 and *p* = 0.0277, respectively; Figures 3(d) and 3(e)). In addition, a significant macrophage accumulation within the abdominal aortic wall was detected by antimacrophage antibody Mac-3 in mice treated with MRS2578 and showed a tendency of increase with the elevation of MRS2578 dosage (*p* < 0.001, Figures 3(c) and 3(f) for quantitative analysis of result). These findings suggested that MRS2578 could promote the expressions/secretions of VCAM-1 and MCP-1 and subsequently induce local infiltration of macrophages in AAA formation.

### 3.4. Effects of MRS2578 on Lipid Profiles and Blood Pressure

**Figure 4:**
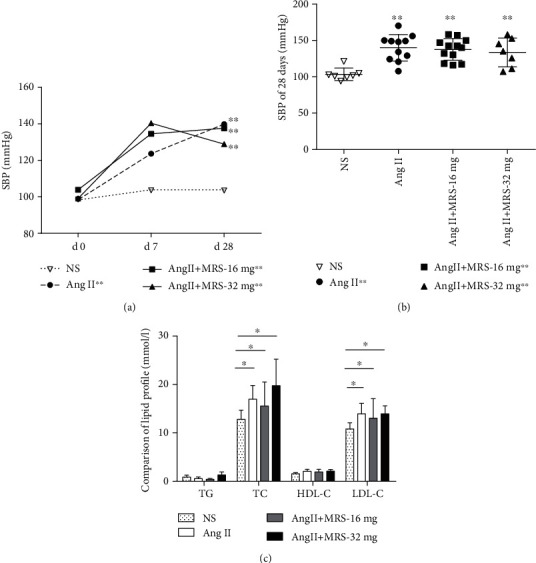
Effects of MRS2578 on the formation of angiotensin II-induced AAA are independent of changes of lipid profiles and blood pressure. (a, b) Infusions of Ang II increase SBP, but coadministration with MRS2578 did not change the blood pressure (*n* = 8~12, ^∗∗^*p* < 0.01 vs. NS group). (c) Lipid profile of each mouse group at 28 days of Ang II infusion (*n* = 8~12, ^∗^*p* < 0.05 vs. the NS group). Data are mean ± SEM.

Stimulation of Ang II could increase blood pressure of mice through several effects including enhanced sympathetic nervous system activity, vasoconstriction, and secretion of aldosterone. As shown in Figure 4(a), systolic blood pressures (SBP) were not different in apoE^−/−^ mice coadministered MRS2578 compared with Ang II, which is consistent with previous results demonstrating that SBP were increased after 28 days of Ang II infusion (Figure 4(b)). After 4 weeks, all mice infused with Ang II exhibited similar increases in blood pressure. We also examined the degree of hyperlipidemia in apoE^−/−^ mice infused with Ang II with or without MRS2578. As expected, the infusion of Ang II induced marked hyperlipidemia. However, intergroup comparisons showed no difference in TC and LDL-C change between the MRS2578-treated group and the vehicle group (Figure 4(c)).

## 4. Discussion

The pathogenesis of AAA is complex and involved in multiple processes. Exploring the potential mechanism of AAA formation and expansion will provide new clues for AAA treatment and prevention. In the present study, the extensively characterized Ang II-induced AAA experimental model in apoE^−/−^ mice was chosen to explore the role of the P2Y_6_ receptor in AAA formation since it has the similar characteristics of AAA in humans [18, 19, 24]. It was firstly found that MRS2578, a selective antagonist of the P2Y_6_ receptor, has a deteriorating effect on Ang II-induced AAA in apoE^−/−^ mice accompanying with increased mortality in the early stage. Histological and morphological examinations of MRS2578-cotreated apoE^−/−^ mice showed a noticeable increase in the characteristic features of AAA including vascular inflammation, disruption of elastin fibers, and aortic dilation. This finding is consistent with Garcia et al. [16] who demonstrated that whole body deficiency in the P2Y_6_ receptor indeed promoted the formation of AAA in Ang II-infused LDLR knockout mice. However, they did not report any difference in mortality. Until now, the underlying mechanisms of blocking the P2Y_6_ receptor exhibit high susceptibility to AAA rupture and had not been fully elucidated.

Chronic inflammation of the vessel wall is considered to be initially and constantly responsible for aneurysmal degeneration and expansion [9, 25, 26] . It is demonstrated that infiltration and accumulation of macrophages in the aorta wall are early events in the apoE^−/−^ mouse model and human AAA [27–30]. Whether through cytokine inhibition or macrophage depletion, reduced macrophage accumulation in the aneurysm wall leads to lower MMP activity and attenuates aneurysm formation in mouse models of AAA [28, 29]. Similarly, we also found macrophage accumulation in the media and adventitia of AAA mice in this study, which is more significant in MRS2578-treated groups. What is more, the role of the intima in AAA formation was explored recently. It has been shown that vascular endothelial inflammation is an earlier event or signal before macrophage infiltration in the process of AAA formation [30]. Activated aortic endothelial cells can generate large numbers of adhesion molecules such as VCAM-1 and MCP-1 that regulate attachment and recruit chemotaxis migration of peripheral monocytes to the aortic wall and result in macrophage infiltration and secondary vascular inflammation in the adventitia and/or media [31–33]. In this study, a high dosage of MRS2578 exhibited upregulation of VCAM-1 in plasma and endothelial cells and local macrophage infiltration, which no doubt contributes to the development of AAA.

In addition to accumulation of macrophages, macrophage polarization plays a crucial role in the development of AAA [34, 35]. M1 macrophages are considered to be proinflammatory; in contrast, M2 macrophages have anti-inflammatory roles [36, 37]. There is a higher M1/M2 ratio in the aorta at the early stage of the Ang II-induced AAA model, associated with a predominant infiltration of macrophages and increased aortic dilatation [35]. A study from Aono et al. showed that Ang II induces macrophage polarization to proinflammatory M1 state via stimulation of the Ang II type 1 receptor (AT1R) [38]. And the correlation of the P2Y_6_ receptor with AT1R is supported by recent data demonstrating that formation of heterodimerization of AT1R and P2Y_6_ receptor can amplify Ang II signaling [17]. Inversely, disruption of the AT1R-P2Y_6_R heterodimer by MRS2578 inhibits Ang II-induced hypertension. However, the pathogenesis of Ang II-induced hypertension is different from that of Ang II-induced AAA. Hence, we cannot ignore the notion that after binding to Ang II, heterodimerization of AT1R and P2Y_6_ receptor may generate a diverse function under different pathogenetic conditions, such as AAA [39]. Therefore, further research should investigate the contributions of MRS2578 on macrophage polarization in vitro and impacts of heterodimerization of AT1R and P2Y_6_ receptor under Ang II-induced AAA.

MMP expression and activity, in particular MMP-2 and MMP-9, has been postulated to be a remarkable factor involved in the initiation and the progression of AAA [40, 41]. MMP-2 or MMP-9 knockout mice exhibit a protective effect in the development of AAA [41]. Numerous reports have documented that MMPs, predominantly produced by neutrophils or macrophages, upregulated in experimental models and in humans and accounted partly for aneurysm formation via leading to the destruction of ECM. Evidence from previous studies has indicated that degeneration of ECM and apoptosis proteins are key events that lead to weakening and dilation of the aortic wall. The deficiency of ECM which maintained aortic wall integrity promotes susceptibility to aneurysm formation and rupture [40]. In the present study, EVG staining and immunohistochemical staining showed that levels of MMP-2 and MMP-9 increased in the media of aortas in the MRS2578 injection group compared with the control group, resulting in the degradation of ECM. Besides, gelatin zymography was used to measure activity of MMP-2 and MMP-9 in the wall of aortic aneurysms. It demonstrated that administration of MRS2578 has potential to directly modulate the expression and activities of MMP-2 and MMP-9 in a dose-dependent manner followed by elastic fiber degradation and disruption of media, which may explain the higher AAA rupture-related mortality in the MRS2578-cotreated group. Taken together, these data corroborate that increased expression and activity of MMP-2 and MMP-9 and subsequently ECM fragmentation are a part of the mechanisms of MRS2578 in promoting formation of AAA.

P2 purinergic receptors are involved in a series of crucial physiological functions [42]. The relationship between P2Y_6_ receptor and inflammation has been extensively explored and is controversial [43–49]. In most cases, activation of the P2Y_6_ receptor would promote inflammatory response in macrophages [43–45]. Activation of the P2Y_6_ receptor by UDP, a selectively stimulator of the P2Y_6_ receptors, potentiated proinflammatory responses in THP-1 cells in a concentration-dependent manner [44]. Similarly, lipopolysaccharide-stimulated primary peritoneal macrophages from P2Y_6_-deficient mice had a defective response to UDP, indicating that the P2Y_6_ receptor displayed proinflammatory effects in macrophages in vitro [43]. However, most data are available either from stimulation of UDP or LPS in vitro or from stimulation of Ang II in whole body *p2y6* knockout. Our study explored the response of macrophages under the Ang II stimulation with pharmacologic inhibition of the P2Y_6_ receptor by MRS2578 in vivo. Therefore, apparently discordant findings may result from the discrepancies between genetic deletion of the P2Y_6_ receptor and pharmacological inhibition. Beyond that, the P2Y_6_ receptor is widely expressed on the cell surface of intimal endothelial cells [14, 50], medial VSMCs [50, 51], macrophages or T cells [52–54], and perivascular adipocytes [55], which all take part in AAA formation. There are still evidences demonstrating that activation of P2Y_6_ could exert an anti-inflammation role in different kinds of cells [46, 47], such as T cells. For example, Salem et al. [46] found that P2Y_6_ receptor^−/−^ mice exacerbated intestinal inflammation induced by dextran sulfate sodium partly via increased recruitment of Th17/Th1 lymphocytes, whose infiltration contributes to formation of AAA [53, 54]. In another study, the specific absence of the P2Y_6_ receptor on T cells amplified cytokine generation, T cell activation, and subsequently exuberant allergen-induced pulmonary inflammation [47]. And the activation of T cells potentiates a cycle of recruitment of macrophages and matrix destruction [53]. According to the above results, it could be conjectured that the P2Y_6_ receptor might possess intricate and different roles in inflammation, and the ultimate comprehensive effect is to promote the development of AAA.

Another possible explanation for the inconsistency between the previous and present studies is that the P2Y_6_ receptor may also influence AAA formation through some noninflammatory mechanisms. Vascular SMC is the main component of media which accounts for the predominant pathological mechanisms of aneurysm development by modulating the balance of its proliferation and apoptotic process [1, 9]. Infiltrated inflammatory cells can promote apoptosis and phenotypic changes of VSMCs in the media, which leads to the imbalance of the matrix producing and matrix repair capacity of the media [9, 56]. Ultimately, these effects can lead to loss of wall elasticity and rupture of AAA. Published studies suggest that Ang II stimulates SMC migration and proliferation via AT1R [57, 58] and the P2Y_6_ receptor can enhance proliferation and migration of SMCs in vitro [15, 59, 60]. Knockdown of the P2Y_6_ receptor can partially reverse the effect of Ang II on human aortic VSMC proliferation [60]. Additionally, Placet et al. [61] indicated that the P2Y_6_ receptor may contribute to intestinal tumorigenesis by blocking the apoptotic process. Thus, it is possible that enhanced apoptosis of SMC and dampened proliferation of SMC caused by treatment of MRS2578 may contribute to the susceptibility to rupture of aneurysms. Further investigation should be undertaken to verify the role of the P2Y_6_ receptor in apoptosis of SMC.

There are other factors that contribute to the progress of AAA, such as blood pressure and high level of cholesterol. Stimulation of Ang II could increase blood pressure via enhancing vasoconstriction, activity of the sympathetic nervous system, and release of aldosterone [62]. However, the observed difference of systolic blood pressure between Ang II-treated mice and Ang II+MRS2578-cotreated mice in this study was not significant. This finding is contrary to previous studies which have suggested that the whole deletion of the P2Y_6_ receptor or MRS2578 treatment suppressed mean blood pressure induced by Ang II due to the formation of a heterodimer between AT1R and P2Y_6_ receptor [17]. This discrepancy between the two studies is in part due to different dosages of MRS2578 used in vivo. Another possibility is that there may be some pathological differences under Ang II stimulation only and Ang II plus high-fat diet treatment. Thus, the inhibiting effect of MRS2578 on disruption of heterodimerization of AT1R and P2Y_6_ receptor may not be enough to decrease the Ang II together with high-fat diet induced hypertension [63], which does not depend on the Ang II-AT1R signaling pathway. Moreover, dysfunctional lipoprotein metabolism correlated directly with human and animal model AAA formation [64, 65]. At 28 days of Ang II infusion, the lipid profile of the four mouse groups whether coadministrated with MRS2578 or not seems to be at similar levels, indicating that the deleterious effects of MRS2578 on AAA formation are independent of Ang II-induced increases in SBP and hyperlipidemia.

## 5. Conclusion

To summarize, the major finding of the present study is that inhibition of the P2Y_6_ receptor with the antagonist MRS2578 leads to enhanced severity and mortality of Ang II-induced AAA. A high dosage of MRS2578 exhibited upregulation of chemokines, local macrophage infiltration, activation of MMP-2/9, and subsequently ECM fragmentation, indicating that MRS2578 may exacerbate AAA and increase mortality at least in part by activating the VCAM-1/MCP-1-macrophages-MMP-2/9 inflammatory pathway. However, the current study has several limitations. First, though the Ang II-induced AAA experimental model has many features similar to the human disease, it still is not consistent with the exact pathologic conditions in human AAA. Further investigation should focus on other widely used CaCl_2_-induced or elastase-treated models. Second, it should be noted that we only examined the deterioration effects of MRS2578 on AAA formation in vivo but did not explore the potential role of the P2Y_6_ receptor expressed on different vascular cells under MRS2578 treatment in vitro. Further work is needed to assess the exact impact of MRS2578 on different vascular cells to confirm and validate these findings.

## Data Availability

All data generated or analyzed during this study are included in this article.

## References

[1] Sakalihasan N., Limet R., Defawe O. D. (2005). Abdominal aortic aneurysm. *Lancet*.

[2] Hussey K., Siddiqui T., Burton P., Welch G. H., Stuart W. P. (2015). Understanding administrative abdominal aortic aneurysm mortality data. *European Journal of Vascular and Endovascular Surgery*.

[3] Mohan I. V., Stephen M. S. (2013). Peripheral arterial aneurysms: open or endovascular surgery?. *Progress in Cardiovascular Diseases*.

[4] Cheng Z., Zhou Y. Z., Wu Y. (2018). Diverse roles of macrophage polarization in aortic aneurysm: destruction and repair. *Journal of Translational Medicine*.

[5] Maegdefessel L., Spin J. M., Raaz U. (2014). miR-24 limits aortic vascular inflammation and murine abdominal aneurysm development. *Nature Communications*.

[6] Jalalzadeh H., Indrakusuma R., Planken R. N., Legemate D. A., Koelemay M. J., Balm R. (2016). Inflammation as a predictor of abdominal aortic aneurysm growth and rupture: a systematic review of imaging biomarkers. *European Journal of Vascular and Endovascular Surgery*.

[7] Lu H., Rateri D. L., Bruemmer D., Cassis L. A., Daugherty A. (2012). Novel mechanisms of abdominal aortic aneurysms. *Current Atherosclerosis Reports*.

[8] Henderson E. L., Geng Y. J., Sukhova G. K., Whittemore A. D., Knox J., Libby P. (1999). Death of smooth muscle cells and expression of mediators of apoptosis by T lymphocytes in human abdominal aortic aneurysms. *Circulation*.

[9] Golledge J. (2019). Abdominal aortic aneurysm: update on pathogenesis and medical treatments. *Nature Reviews Cardiology*.

[10] Burnstock G. (1972). Purinergic nerves. *Pharmacological Reviews*.

[11] Burnstock G. (2007). Purine and pyrimidine receptors. *Cellular and molecular life sciences : CMLS*.

[12] Liu O., Jia L., Liu X. (2012). Clopidogrel, a platelet P2Y12 receptor inhibitor, reduces vascular inflammation and angiotensin II induced-abdominal aortic aneurysm progression. *PLoS One*.

[13] Dai J., Louedec L., Philippe M., Michel J. B., Houard X. (2009). Effect of blocking platelet activation with AZD6140 on development of abdominal aortic aneurysm in a rat aneurysmal model. *Journal of vascular surgery*.

[14] Riegel A. K., Faigle M., Zug S. (2011). Selective induction of endothelial P2Y6 nucleotide receptor promotes vascular inflammation. *Blood*.

[15] Bar I., Guns P. J., Metallo J. (2008). Knockout mice reveal a role for P2Y6 receptor in macrophages, endothelial cells, and vascular smooth muscle cells. *Molecular Pharmacology*.

[16] Garcia R. A., Yan M., Search D. (2014). P2Y6 receptor potentiates pro-inflammatory responses in macrophages and exhibits differential roles in atherosclerotic lesion development. *PLoS One*.

[17] Nishimura A., Sunggip C., Tozaki-Saitoh H. (2016). Purinergic P2Y6 receptors heterodimerize with angiotensin AT1 receptors to promote angiotensin II-induced hypertension. *Science Signaling*.

[18] Daugherty A., Manning M. W., Cassis L. A. (2000). Angiotensin II promotes atherosclerotic lesions and aneurysms in apolipoprotein E-deficient mice. *The Journal of Clinical Investigation*.

[19] Daugherty A., Manning M. W., Cassis L. A. (2001). Antagonism of AT2 receptors augments angiotensin II-induced abdominal aortic aneurysms and atherosclerosis. *British Journal of Pharmacology*.

[20] Zhang Z., Xu J., Liu Y. (2018). Mouse macrophage specific knockout of SIRT1 influences macrophage polarization and promotes angiotensin II-induced abdominal aortic aneurysm formation. *Journal of Genetics and Genomics*.

[21] Kurtz T. W., Griffin K. A., Bidani A. K., Davisson R. L., Hall J. E. (2005). Recommendations for blood pressure measurement in humans and experimental animals. Part 2: Blood pressure measurement in experimental animals: a statement for professionals from the subcommittee of professional and public education of the American Heart Association council on high blood pressure research. *Hypertension*.

[22] Zhao S. P., Liu L., Cheng Y. C. (2004). Xuezhikang, an extract of cholestin, protects endothelial function through antiinflammatory and lipid-lowering mechanisms in patients with coronary heart disease. *Circulation*.

[23] Dale M. A., Suh M. K., Zhao S. (2015). Background differences in baseline and stimulated MMP levels influence abdominal aortic aneurysm susceptibility. *Atherosclerosis*.

[24] Schriefl A. J., Collins M. J., Pierce D. M., Holzapfel G. A., Niklason L. E., Humphrey J. D. (2012). Remodeling of intramural thrombus and collagen in an Ang-II infusion ApoE-/- model of dissecting aortic aneurysms. *Thrombosis research*.

[25] Sun Y., Zhong L., He X. (2019). LncRNA H19 promotes vascular inflammation and abdominal aortic aneurysm formation by functioning as a competing endogenous RNA. *Journal of Molecular and Cellular Cardiology*.

[26] Sagan A., Mikolajczyk T. P., Mrowiecki W. (2019). T Cells Are Dominant Population in Human Abdominal Aortic Aneurysms and Their Infiltration in the Perivascular Tissue Correlates With Disease Severity. *Frontiers in immunology*.

[27] Saraff K., Babamusta F., Cassis L. A., Daugherty A. (2003). Aortic dissection precedes formation of aneurysms and atherosclerosis in angiotensin II-infused, apolipoprotein E-deficient mice. *Arteriosclerosis, Thrombosis, and Vascular Biology*.

[28] Potteaux S., Tedgui A. (2015). Monocytes, macrophages and other inflammatory mediators of abdominal aortic aneurysm. *Current Pharmaceutical Design*.

[29] Ren J., Liu Z., Wang Q. (2016). Andrographolide Ameliorates Abdominal Aortic Aneurysm Progression by Inhibiting Inflammatory Cell Infiltration through of Cytokine and Integrin Expression. *The Journal of Pharmacology and Experimental Therapeutics*.

[30] Saito T., Hasegawa Y., Ishigaki Y. (2013). Importance of endothelial NF-*κ*B signalling in vascular remodelling and aortic aneurysm formation. *Cardiovascular Research*.

[31] Tieu B. C., Lee C., Sun H. (2009). An adventitial IL-6/MCP1 amplification loop accelerates macrophage-mediated vascular inflammation leading to aortic dissection in mice. *The Journal of Clinical Investigation*.

[32] Hans C. P., Koenig S. N., Huang N. (2012). Inhibition of Notch1 signaling reduces abdominal aortic aneurysm in mice by attenuating macrophage-mediated inflammation. *Arteriosclerosis, Thrombosis, and Vascular Biology*.

[33] Ishibashi M., Egashira K., Zhao Q. (2004). Bone marrow-derived monocyte chemoattractant protein-1 receptor CCR2 is critical in angiotensin II-induced acceleration of atherosclerosis and aneurysm formation in hypercholesterolemic mice. *Arteriosclerosis, Thrombosis, and Vascular Biology*.

[34] Raffort J., Lareyre F., Clément M., Hassen-Khodja R., Chinetti G., Mallat Z. (2017). Monocytes and macrophages in abdominal aortic aneurysm. *Nature Reviews Cardiology*.

[35] Qin Z., Bagley J., Sukhova G. (2015). Angiotensin II-induced TLR4 mediated abdominal aortic aneurysm in apolipoprotein E knockout mice is dependent on STAT3. *Journal of Molecular and Cellular Cardiology*.

[36] Dale M. A., Ruhlman M. K., Baxter B. T. (2015). Inflammatory cell phenotypes in AAAs: their role and potential as targets for therapy. *Arteriosclerosis, Thrombosis, and Vascular Biology*.

[37] Murray P. J., Allen J. E., Biswas S. K. (2014). Macrophage activation and polarization: nomenclature and experimental guidelines. *Immunity*.

[38] Aono J., Suzuki J., Iwai M. (2012). Deletion of the angiotensin II type 1a receptor prevents atherosclerotic plaque rupture in apolipoprotein E-/- mice. *Arteriosclerosis, Thrombosis, and Vascular Biology*.

[39] Sunggip C., Nishimura A., Shimoda K., Numaga-Tomita T., Tsuda M., Nishida M. (2017). Purinergic P2Y_6_ receptors: a new therapeutic target of age-dependent hypertension. *Pharmacological Research*.

[40] Freestone T., Turner R. J., Coady A., Higman D. J., Greenhalgh R. M., Powell J. T. (1995). Inflammation and matrix metalloproteinases in the enlarging abdominal aortic aneurysm. *Arteriosclerosis, Thrombosis, and Vascular Biology*.

[41] Longo G. M., Xiong W., Greiner T. C., Zhao Y., Fiotti N., Baxter B. T. (2002). Matrix metalloproteinases 2 and 9 work in concert to produce aortic aneurysms. *The Journal of Clinical Investigation*.

[42] Idzko M., Ferrari D., Eltzschig H. K. (2014). Nucleotide signalling during inflammation. *Nature*.

[43] Kukulski F., Ben Yebdri F., Lefebvre J., Warny M., Tessier P. A., Sévigny J. (2007). Extracellular nucleotides mediate LPS-induced neutrophil migration in vitro and in vivo. *Journal of leukocyte biology*.

[44] Warny M., Aboudola S., Robson S. C. (2001). P2Y(6) nucleotide receptor mediates monocyte interleukin-8 production in response to UDP or lipopolysaccharide. *The Journal of biological chemistry*.

[45] Stachon P., Peikert A., Michel N. A. (2014). P2Y6Deficiency limits vascular inflammation and atherosclerosis in mice. *Arteriosclerosis, Thrombosis, and Vascular Biology*.

[46] Salem M., El Azreq M. A., Pelletier J., Robaye B., Aoudjit F., Sévigny J. (2019). Exacerbated intestinal inflammation in P2Y_6_ deficient mice is associated with Th17 activation. *Biochimica et biophysica acta Molecular basis of disease*.

[47] Giannattasio G., Ohta S., Boyce J. R., Xing W., Balestrieri B., Boyce J. A. (2011). The purinergic G protein-coupled receptor 6 inhibits effector T cell activation in allergic pulmonary inflammation. *The Journal of Immunology*.

[48] Huang D., Yang J., Liu X. (2018). P2Y_6_ receptor activation is involved in the development of neuropathic pain induced by chronic constriction injury of the sciatic nerve in rats. *Journal of Clinical Neuroscience*.

[49] Bernier L. P., Ase A. R., Boué-Grabot É., Séguéla P. (2013). Inhibition of P2X4 function by P2Y6 UDP receptors in microglia. *Glia*.

[50] Wang L., Karlsson L., Moses S. (2002). P2 receptor expression profiles in human vascular smooth muscle and endothelial cells. *Journal of Cardiovascular Pharmacology*.

[51] Chang K., Hanaoka K., Kumada M., Takuwa Y. (1995). Molecular cloning and functional analysis of a novel P2 nucleotide receptor. *The Journal of biological chemistry*.

[52] Somers G. R., Hammet F. M., Trute L., Southey M. C., Venter D. J. (1998). Expression of the P2Y6 purinergic receptor in human T cells infiltrating inflammatory bowel disease. *Laboratory Investigation*.

[53] Ocana E., Bohórquez J.-C., Pérez-Requena J., Brieva J. A., Rodríguez C. (2003). Characterisation of T and B lymphocytes infiltrating abdominal aortic aneurysms. *Atherosclerosis*.

[54] Shimizu K., Mitchell R. N., Libby P. (2006). Inflammation and cellular immune responses in abdominal aortic aneurysms. *Arteriosclerosis, Thrombosis, and Vascular Biology*.

[55] Burnstock G., Gentile D. (2018). The involvement of purinergic signalling in obesity. *Purinergic Signal*.

[56] Zhang S. L., Du X., Chen Y. Q., Tan Y. S., Liu L. (2018). Potential medication treatment according to pathological mechanisms in abdominal aortic aneurysm. *Journal of Cardiovascular Pharmacology*.

[57] Valente A. J., Yoshida T., Wekerle R., Katsuyama M., Chandrasekar B. (2012). Physical association of angiotensin-II type 1 receptor (AT1R) and NOX-1 mediates NF-*κ*B and AP-1-dependent interleukin- 18 induction and aortic SMC migration and proliferation. *The FASEB Journal*.

[58] Won S.-M., Park Y.-H., Kim H.-J., Park K.-M., Lee W.-J. (2006). Catechins inhibit angiotensin II-induced vascular smooth muscle cell proliferation via mitogen-activated protein kinase pathway. *Experimental & Molecular Medicine*.

[59] Hou M., Harden T. K., Kuhn C. M. (2002). UDP acts as a growth factor for vascular smooth muscle cells by activation of P2Y(6) receptors. *American Journal of Physiology Heart and Circulatory Physiology*.

[60] Wang S., Tang L., Zhou Q. (2017). miR-185/P2Y6 Axis inhibits angiotensin II-induced human aortic vascular smooth muscle cell proliferation. *DNA and Cell Biology*.

[61] Placet M., Arguin G., Molle C. M. (2018). The G protein-coupled P2Y6 receptor promotes colorectal cancer tumorigenesis by inhibiting apoptosis. *Biochimica et biophysica acta Molecular basis of disease*.

[62] Te Riet L., van Esch J. H. M., Roks A. J. M., van den Meiracker A. H., Danser A. H. J. (2015). Hypertension: renin-angiotensin-aldosterone system alterations. *Circulation Research*.

[63] Wang Y., Song Y., Suo M., Jin X., Tian G. (2012). Telmisartan prevents high-fat diet-induced hypertension and decreases perirenal fat in rats. *Journal of Biomedical Research*.

[64] Delbosc S., Diallo D., Dejouvencel T. (2013). Impaired high-density lipoprotein anti-oxidant capacity in human abdominal aortic aneurysm. *Cardiovascular Research*.

[65] Harrison S. C., Holmes M. V., Burgess S. (2018). Genetic association of lipids and lipid drug targets with abdominal aortic aneurysm: a meta-analysis. *JAMA Cardiology*.

