# Erratum to “The Role of a Selective P2Y_6_ Receptor Antagonist, MRS2578, on the Formation of Angiotensin II-Induced Abdominal Aortic Aneurysms”

**DOI:** 10.1155/2020/8569421

**Published:** 2020-09-26

**Authors:** Xiao Du, Shilan Zhang, Qunyan Xiang, Jingyuan Chen, Feng Tian, Jin Xu, Xin Li, Yuansheng Tan, Ling Liu

**Affiliations:** ^1^Department of Cardiovascular Medicine, The Second Xiangya Hospital, Central South University, Changsha, Hunan 410011, China; ^2^Department of Gastroenterology, The Second Xiangya Hospital, Central South University, Changsha, Hunan 410011, China; ^3^Ministry of Education, Hospital of Hunan University of Traditional Chinese Medicine, Changsha, Hunan 410011, China

In the article titled “The Role of a Selective P2Y_6_ Receptor Antagonist, MRS2578, on the Formation of Angiotensin II-Induced Abdominal Aortic Aneurysms” [1], there was an error in affiliation numbers 1 and 2. The address line of “The Second Xiangya Hospital” was missing in both affiliations. This error was introduced during the production of the article, and the publisher apologises for this error. The corrected affiliations appear in the affiliation list.
